# Supplementary material for: Gastric proton pump with two occluded K+ engineered with sodium pump-mimetic mutations
Source: Nat Commun. 2021 Sep 29;12:5709. doi: 10.1038/s41467-021-26024-1 (PMC8481561; doi:10.1038/s41467-021-26024-1)
Supplement: Supplementary file 1 — Supplementary Information [file 41467_2021_26024_MOESM1_ESM.pdf]

Supplementary Information

Gastric proton pump with two occluded K^+ engineered with sodium pump-mimetic mutations

Kazuhiro Abe,[1,2*] Kenta Yamamoto,[1,2] Katsumasa Irie,[3] Tomohiro Nishizawa,[4] Atsunori Oshima[1,2]

[1]Cellular and Structural Physiology Institute, Nagoya University, 464-8601, Japan

[2]Graduate School of Pharmaceutical Sciences, Nagoya University, 464-8601, Japan

[3]Department of Biophysical Chemistry, Faculty of Pharmaceutical Sciences, Wakayama Medical University, Wakayama, 25-1 Shichibancho, 640-8156, Japan

[4]Graduate School of Medical Life Science, Yokohama City University, Tsurumi, Yokohama, 230-0045, Japan

*Correspondence: Kazuhiro Abe, kabe@cespi.nagoya-u.ac.jp

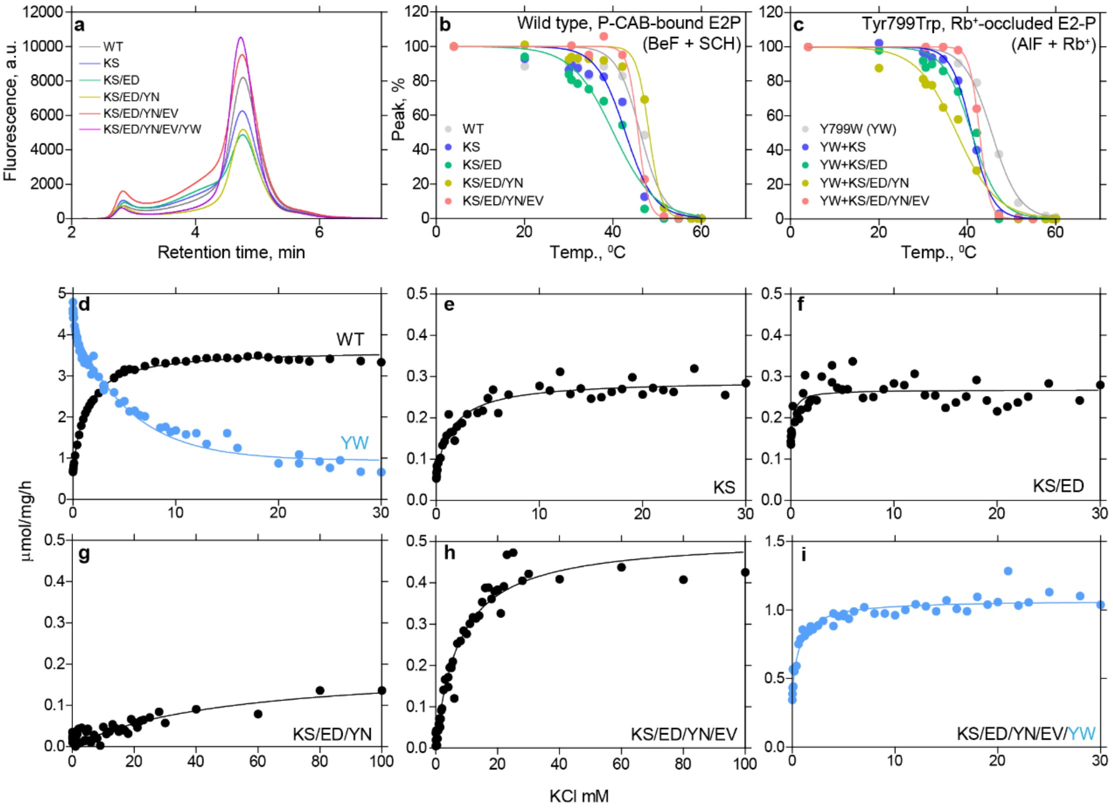

Supplementary Fig 1| Thermal stabilities and K^+-dependent ATPase activities of HKA mutants **a**, the expression levels of each mutant in their membrane fractions were determined by fluorescence size-exclusion chromatography (FSEC). Membrane fractions were solubilized by LMNG in the presence of BeF and monitored by their GFP fluorescence. Peak value indicates relative contents of HKA wild type or mutants in the membrane fractions. **b**,**c**, thermal stabilities of wild type and indicated mutants of HKA in the presence of BeF and SCH28080 (**b**) or AlF and 100 mM Rb^+ (**c**) were determined as described in the Methods. Peak values in the FSEC analysis are plotted as a function of treatment temperature. Peak value of the sample kept on ice (0°C) was set as 100%, and the temperature giving 50% peak intensity was taken as T_m (Supplementary Table 1). Individual data from a single experiment at different temperatures are plotted. Note, we employ Tyr799Trp mutant (YW) as a base construct for the K^+-occluded forms evaluated in **c** (15). **d-h**, K^+-dependent ATPase activity in the membrane fractions as described in the Methods. Data were fitted to the Michalis-Menten equation with a background H^+-ATPase activity measured at 0 mM KCl. Only mutants having Tyr799Trp (YW) are highlighted in blue, while others are in black. The ATPase activity in the presence of 10 µM vonoprazan does not change over the evaluated concentrations of K^+, and was set as a blank value. V_{max} and $K_{0.5,K+}$ are displayed in Supplementary Table 2. Individual data taken at indicated K^+-concentrations are plotted. Representative results from more than three independent measurements for each of the mutants are shown in the figure.

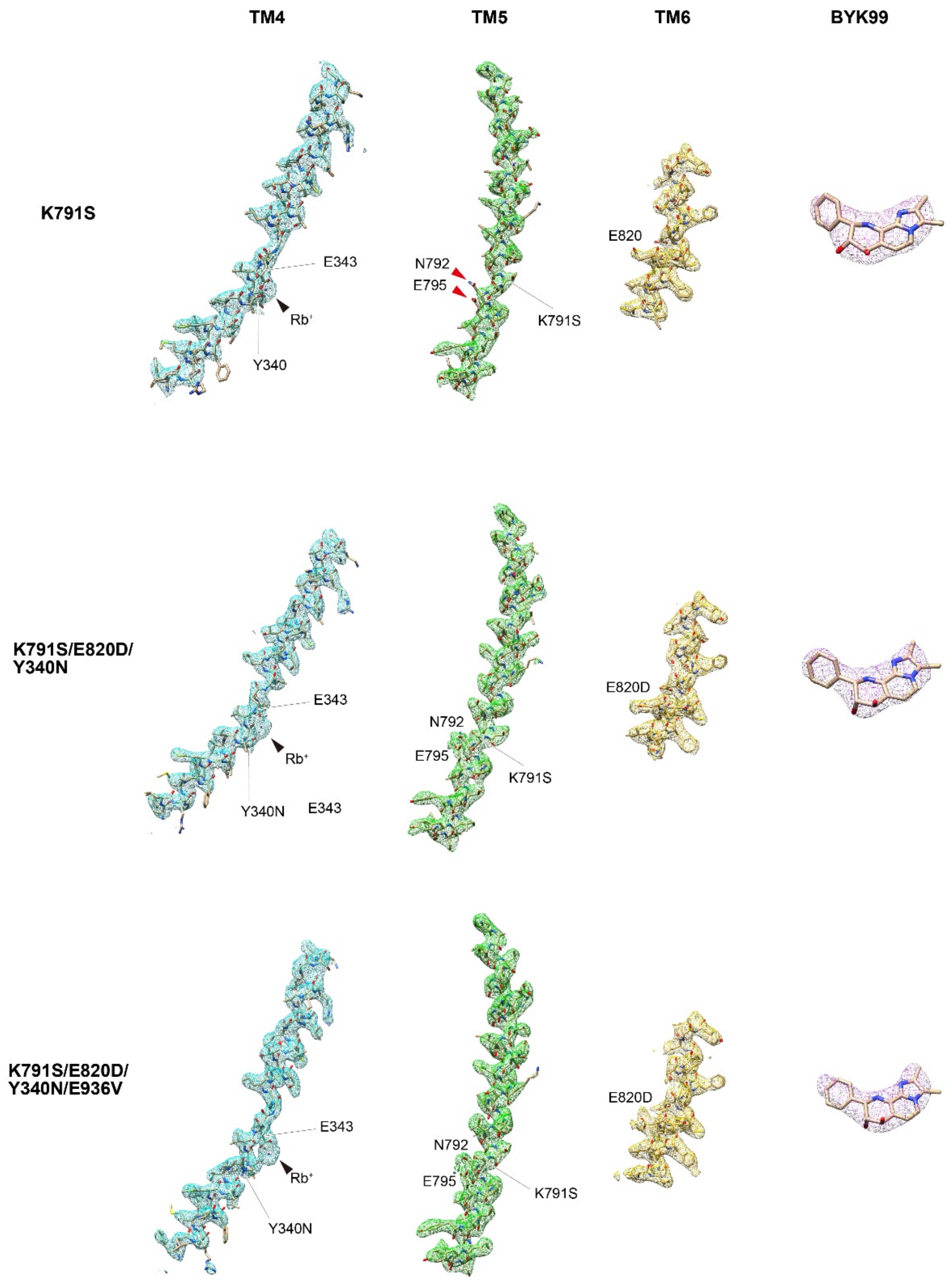

Supplementary Fig 2| Electron density maps for the TM helices and bound BYK99 of HKA mutants

Mesh represents 2Fo-Fc electron density maps around the indicated portion of the mutant enzymes contoured at 2σ level. Black arrowheads indicate Rb^+ at the cation-binding site. Red arrowheads indicate residues with poor density in the KS single mutant.

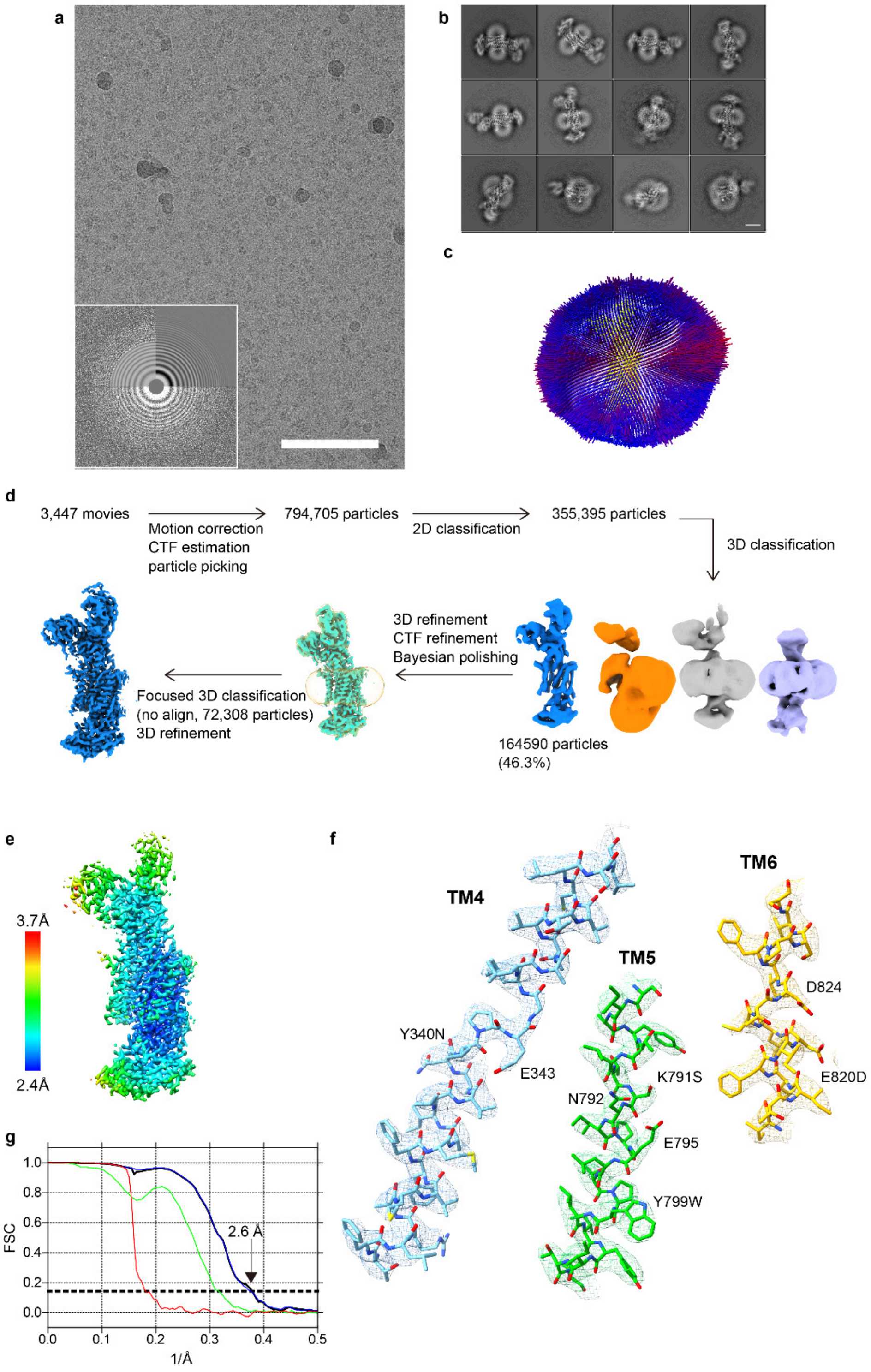

Supplementary Fig 3| Cryo-EM analysis of HKA quintuple mutant
a, a representative cryo-EM image and its Fourier transform. Bar, 50 nm. **b**, representative 2D-class averages. Bar, 5 nm. **c**, Angular distribution plot of particles included in the final 3D reconstruction. The number of views at each angular orientation is represented by the length and color of cylinders. Red indicates more views. **d**, Data processing (see Methods). **e**, Final reconstruction map colored by local resolution as calculated by RELION3.1. **f**, EM density maps of indicated regions (7σ). **g**, FSC plot used for resolution estimations (black: corrected, blue: masked, green: unmasked, red: phase randomized).

Supplementary Table 1| Thermal stabilities of HKA mutants

Thermal stabilities (T_m) of mutants evaluated in this study are summarized. Values were calculated from data shown in Fig. 2. See Methods for details. Analyzed resolution of the crystal structures and the PDB codes of indicated mutants are shown.

[1]N-terminal deleted construct is referred to as WT.

[2]Note, we employ Tyr799Trp mutant (YW) that prefers the K^+-occluded state as a base construct for the K^+-occluded forms.

[3]No crystals obtained.

[4]Crystal diffracted to around 20Å. [5]Resolution obtained by Cryo-EM analysis

Base construct, conformation	Additional mutation	T_m, °C	resolution, Å	PDB ID
WT[1], (SCH)E2BeF	-	46.2	2.8	5YLU
	KS	42.8	3.4	7EFL
	KS/ED	40.1	4.5	-
	KS/KD/YN	48.2	3.2	7EFM
	KS/ED/YN/EV	46.7	3.2	7EFN
Y799W[2], (K^+)E2-AlF	-	45.5	2.5	6JXH
	KS	41.0	-[3]	-
	KS/ED	41.0	-	-
	KS/KD/YN	37.6	-	-
	KS/ED/YN/EV	42.8	~20[4] (2.6)[5]	7ET1 (EMD-31294)

Supplementary Table 2| Normalized ATPase activities of mutant enzymes

The maximum values of K^+-dependent ATPase activities in the membrane fractions prepared from cells expressing indicated mutants are shown (V_{max}, µmol/mg/h). The expression levels in the membrane fractions (injected approximately 20 µg of membrane fraction) were determined by monitoring the fluorescence of N-terminus GFP-tag separated by FSEC, and normalized per 1 mg of membrane fraction (Peak/mg, in a.u.). Normalized ATPase activities were then obtained as their activity per detected fluorescence, and compared to the WT (as 100%). Apparent K^+-affinities for the ATPase activity are also indicated ($K_{0.5,K+}$). Values were calculated from data shown in Supplementary Fig. 1A (Peak/mg) and Supplementary Fig. 1D-I (V_{max}, $K_{0.5,K+}$).

Construct	Peak/mg	V_{max}, µmol/mg/h	$K_{0.5,K+}$, mM	Normalized V_{max}, % of WT
WT	8200	3.5	1.25	100
KS	6200	0.28	1.63	10.6
KS/ED	4800	0.26	0.26	5.9
KS/ED/YN	5200	0.20	50.0	9.0
KS/ED/YN/EV	9500	0.51	7.62	12.6
KS/ED/YN/EV/YW	10500	1.09	0.77	24.3

Supplementary Table 3| Data collection and refinement statistics

[1]Statistics for the highest-resolution shell are shown in parentheses.

[2]The diffraction data are anisotropic.

	KS	KS (Rb^+)	KS/ED	KS/ED (Rb^+)	KS/ED/YN	KS/ED/YN (Rb^+)	KS/ED/YN/EV	KS/ED/YN/EV (Rb^+)	Wild type (Rb^+)
PDB ID	7EFL				7EFM		7EFN		
Data collection									
Wave length (Å)	1.00	0.814	1.00	0.814	1.00	0.814	1.00	0.814	0.814
Resolution (Å)	46-3.4 (3.5-3.4)[1]	48-3.7 (3.8-3.7)	48-4.5 (4.7-4.5)	48-4.5 (4.7-4.5)	48-3.2 (3.3-3.2)	48-3.2 (3.3-3.2)	46-3.2 (3.3-3.2)	48-4.0 (4.1-4.0)	48-3.4 (3.5-3.4)
Space group	$P\,3_1\,2\,1$	$P\,3_1\,2\,1$	$P\,3_1\,2\,1$	$P\,3_1\,2\,1$	$P\,3_1\,2\,1$	$P\,3_1\,2\,1$	$P\,3_1\,2\,1$	$P\,3_1\,2\,1$	$P\,3_1\,2\,1$
Cell dimension									
a, *b*, *c* (Å)	105.0,105.0,369.9	104.5, 104.5, 367.2	103.9, 103.9, 368.3	103.9, 103.9, 368.3	104.7, 104.7, 368.0	104.7, 104.7, 368.0	105.9, 105.9, 372.0	105.3, 105.3, 370.9	106.1, 106.1, 371.6
α, β, γ (°)	90, 90, 120	90, 90, 120	90, 90, 120	90, 90, 120	90, 90, 120	90, 90, 120	90, 90, 120	90, 90, 120	90, 90, 120
R_{merge}	0.165 (2.48)	0.032 (0.95)	0.017 (0.42)	0.018 (1.35)	0.11 (2.58)	0.033 (1.35)	0.033 (2.60)	0.027 (0.51)	0.034 (0.44)
R_{pim}	0.039 (0.548)	0.032 (0.95)	0.017 (0.42)	0.018 (1.35)	0.026 (0.57)	0.033 (1.35)	0.033 (2.60)	0.027 (0.51)	0.034 (0.44)
$I / \sigma I$	12.4 (1.35)	10.8 (0.74)	11.4 (1.91)	9.13 (0.71)	16.8 (1.6)	11.0 (0.54)	9.15 (0.28)	8.99 (1.07)	15.7 (1.44)
$CC_{1/2}$	0.99 (0.79)	1 (0.40)	1 (0.95)	1 (0.62)	0.99 (0.85)	1 (0.32)	1 (0.16)	1 (0.90)	1 (0.72)
Completeness (%)	99.8 (99.8)	85.7 (13.1)	98.3 (98.0)	98.3 (98.0)	94.8 (56.6)	94.8 (56.6)	81.7 (25.2)[2]	98.6 (96.8)	99.2 (97.5)
Redundancy	20.0 (21.1)	2.0 (2.0)	2.0 (2.0)	2.0 (2.0)	20.1 (21.4)	2.0 (2.0)	2.0 (2.0)	2.0 (2.0)	2.0 (2.0)
**Refinement**									
No. of reflections	33564 (3274)		14176 (1358)		39699 (2203)		41073 (1017)		
R_w/R_f (%)	23.0/28.9 (32.2/35.0)		26.1/37.7 (30.2/43.9)		22.8/29.1 (31.4/37.7)		27.1/30.7 (36.8/38.9)		
Wilson *B*-factor	94.6		240		82.1		58.03		
No. of atoms	9756		9736		9816		9844		
Protein	9697		9736		9747		9733		
Ligands	59		-		69		111		
Average *B*-factor	101		287		83.5		60.1		
Protein ($Å^2$)	101.1		287		83.2		60.0		
Ligands ($Å^2$)	97.9		-		113.9		68.2		
R.m.s. deviations									
Bond length (Å)	0.012		0.02		0.01		0.004		
Bond angles (°)	1.34		2.26		1.33		0.88		
Validation									
MolProbity score	2.2		2.51		2.13		1.65		
Clashscore	20.7		23.3		18.5		10.1		
Poor rotamers (%)	0.2		0.2		0.1		0.0		
Ramachandran plot									
Favored (%)	93.4		85.3		94.8		97.3		
Allowed (%)	6.6		14.3		5.2		2.7		
Outliers (%)	0.0		0.4		0.0		0.0		

Supplementary Table 4| Cryo-EM data collection, processing, refinement and validations

	KS/ED/YN/EV/YW (EMD-31294) (PDB 7ET1)
**Data collection and processing**	
Magnification	105,000
Voltage (kV)	300
Electron exposure (e–/Å^2)	48
Defocus range (μm)	0.8 – 1.8
Pixel size (Å)	0.83
Symmetry imposed	*C1*
Initial particle images (no.)	794,705
Final particle images (no.)	72,308
Map resolution (Å)	2.6
FSC threshold	0.143
Map resolution range (Å)	3.7-2.4
**Refinement**	
Initial model used (PDB code)	6JXH
Model resolution (Å)	2.9
FSC threshold	0.5
Model resolution range (Å)	80-2.6
Map sharpening *B* factor (Å^2)	-47.4
Model composition	
Non-hydrogen atoms	10029
Protein residues	1249
Ligands	1 Water,3 K^+,1 Mg^{2+},1 AlF,2 PCW, 1 CLR
B factors (Å^2)	
Protein	21.99
Ligand	32.58
Water	33.53
R.m.s. deviations	
Bond lengths (Å)	0.005
Bond angles (°)	0.771
Validation	
MolProbity score	1.86
Clashscore	8.65
Poor rotamers (%)	2.16
Ramachandran plot	
Favored (%)	97.3
Allowed (%)	2.73
Disallowed (%)	0.00
CaBLAM scores	
Outliers (%)	1.37
Disfavored (%)	5.48
Cα outliers (%)	0.32

Supplementary Table 5| Coordination geometry and partial valence in the K^+-binding site of HKA and NKA

Partial valence was calculated for K^+ in each of the corresponding structures. Only oxygen atoms within 4Å of K^+ were included for the valence calculation (35,36). See Methods for more details.

PDB<br>Protein<br>State	7ET1 (present study)<br>HKA quintuple mutant<br>$(K^+)_2$E2-AlF				2ZXE (ref. 29)<br>NKA wild type<br>$(K^+)_2$E2-MgF		
Site	Amino acids	Atom	Distance	Valence	Distance	Valence	
I	Thr788	O	2.90	0.11	2.73	0.19	(Thr779 O)
	Lys791Ser	Oγ	2.70	0.21	2.79	0.16	(Ser782 Oγ)
	Asn792	Oδ1	2.79	0.16	2.87	0.12	(Asn783 Oδ)
	Glu795	Oε1	3.74	0.01	3.88	0.01	(Glu786 Oε1)
		Oε2	3.92	0.01			
	Glu820Asp	Oδ1	2.93	0.10	3.15	0.05	(Asp811 Oδ1)
					2.67	0.23	(Asp811 Oδ2)
	Asp824	Oδ1	3.13	0.06			
					2.6	0.30	(H_2O 001)
**Total valence**				**0.65**		**1.06**	
II	Val338	O	3.09	0.06	3.00	0.08	(Val329 O)
	Ala339	O	2.98	0.09	3.04	0.07	(Ala330 O)
	Val341	O	2.73	0.19	2.76	0.17	(Val332 O)
	Glu343	Oε1	3.38	0.03	3.35	0.03	(Glu334 Oε1)
		Oε2					
	Asn792	Oδ1	3.21	0.04	3.07	0.07	(Asn783 Oδ1)
	Glu795	Oε1	2.94	0.10	2.91	0.11	(Glu786 Oε1)
	Glu820Asp	Oδ1	2.82	0.14	3.77	0.01	(Asp811 Oδ1)
		Oδ2	3.40	0.03	2.94	0.1	(Asp811 Oδ2)
**Total valence**				**0.68**		**0.64**	
