# Supplementary material for: Gastric proton pump with two occluded K+ engineered with sodium pump-mimetic mutations
Source: Nat Commun. 2021 Sep 29;12:5709. doi: 10.1038/s41467-021-26024-1 (PMC8481561; doi:10.1038/s41467-021-26024-1)
Supplement: Supplementary file 7 — Description of Additional Supplementary Files [file 41467_2021_26024_MOESM7_ESM.pdf]

Description of Additional Supplementary Files

Supplementary Movie 1| Transmembrane cation-binding site of the KS/ED/YN triple mutant

Structure of the cation-binding site in the KS/ED/YN triple mutant in BYK·Rb^+·E2BeF form (7EFM, color codes as in Fig. 3) in ribbon representations, viewed from approximately parallel to the membrane plane with cytoplasmic-side up. For comparison, HKA WT in SCH·Rb^+·E2BeF (5YLV, wheat) and NKA bufalin·2K^+·E2P (4RES, grey) were superimposed as in Fig. 3. Dotted lines represent expected polar interactions between atoms, except Lys791Ser-Asp824 (4.8 Å). Only side chains discussed in this study are shown as sticks. Rb^+ or K^+ ions (7EFM, purple; 5YLV, wheat; 4RES, dark grey) are shown as small spheres.

Supplementary Movie 2| Transmembrane cation-binding site of the KS/ED/YN/EV quadruple mutant

Structure of the cation-binding site in the KS/ED/YN/EV quadruple mutant in BYK·Rb^+·E2BeF form (7EFN, color codes as in Fig. 3) in ribbon representations, viewed from approximately parallel to the membrane plane with cytoplasmic-side up. For comparison, HKA WT in SCH·Rb^+·E2BeF (5YLV, wheat) and NKA bufalin·2K^+·E2P (4RES, grey) were superimposed as in Fig. 3. Dotted lines represent expected polar interactions between atoms, except Lys791Ser-Asp824 (4.7 Å). Only side chains discussed in this study are shown as sticks. Rb^+ or K^+ ions (7EFN, purple; 5YLV, wheat; 4RES, dark grey) are shown as small spheres.

Supplementary Movie 3| EM density map of the KS/ED/YN/EV/YW quintuple mutant

The EM density map (blue transparent surface, contoured at 7σ) of the KS/ED/YN/EV/YW quintuple mutant of HKA in $(K^+)_2$E2-AlF form are shown with superimposed amino acid model (sticks, color codes as in Fig. 5, 7ET1), viewed from approximately parallel to the membrane plane with cytoplasmic-side up. Occluded two K^+ ions at sites I and II (purple small spheres) and coordinating oxygen atoms are connected with dotted lines (within 4 Å).

Supplementary Movie 4| Transmembrane cation-binding site of the KS/ED/YN/EV/YW quintuple mutant

Structure of the cation-binding site in the KS/ED/YN/EV/YW quintuple mutant in $(K^+)_2$E2-AlF form (7ET1, color codes as in Fig. 5) in ribbon representations, viewed from approximately parallel to the membrane plane with cytoplasmic-side up. For comparison, HKA WT in (K^+)E2-MgF (6JXH, wheat) and NKA $(K^+)_2$E2-MgF (2ZXE, grey) were superimposed as in Fig. 5. Dotted lines represent expected polar interactions between atoms, and now Lys791Ser and Asp824 are within a hydrogen-bond distance (3.0 Å). Only side chains discussed in this study are shown as sticks. K^+ ions (7ET1, purple; 6JXH, wheat; 2ZXE, dark grey) are shown as small spheres.
