# Supplementary material for: Gastric proton pump with two occluded K+ engineered with sodium pump-mimetic mutations
Source: Nat Commun. 2021 Sep 29;12:5709. doi: 10.1038/s41467-021-26024-1 (PMC8481561; doi:10.1038/s41467-021-26024-1)
Supplement: Supplementary file 8 — Reporting Summary [file 41467_2021_26024_MOESM8_ESM.pdf]

nature portfolio

Corresponding author(s): Kazuhiro Abe

Last updated by author(s): Aug 27, 2021

# Reporting Summary

Nature Portfolio wishes to improve the reproducibility of the work that we publish. This form provides structure for consistency and transparency in reporting. For further information on Nature Portfolio policies, see our Editorial Policies and the Editorial Policy Checklist.

## Statistics

For all statistical analyses, confirm that the following items are present in the figure legend, table legend, main text, or Methods section.

| n/a | Confirmed | |
|---|---|---|
| ☒ | ☐ | The exact sample size (*n*) for each experimental group/condition, given as a discrete number and unit of measurement |
| ☒ | ☐ | A statement on whether measurements were taken from distinct samples or whether the same sample was measured repeatedly |
| ☒ | ☐ | The statistical test(s) used AND whether they are one- or two-sided<br>*Only common tests should be described solely by name; describe more complex techniques in the Methods section.* |
| ☒ | ☐ | A description of all covariates tested |
| ☒ | ☐ | A description of any assumptions or corrections, such as tests of normality and adjustment for multiple comparisons |
| ☒ | ☐ | A full description of the statistical parameters including central tendency (e.g. means) or other basic estimates (e.g. regression coefficient) AND variation (e.g. standard deviation) or associated estimates of uncertainty (e.g. confidence intervals) |
| ☒ | ☐ | For null hypothesis testing, the test statistic (e.g. *F*, *t*, *r*) with confidence intervals, effect sizes, degrees of freedom and *P* value noted<br>*Give P values as exact values whenever suitable.* |
| ☒ | ☐ | For Bayesian analysis, information on the choice of priors and Markov chain Monte Carlo settings |
| ☒ | ☐ | For hierarchical and complex designs, identification of the appropriate level for tests and full reporting of outcomes |
| ☒ | ☐ | Estimates of effect sizes (e.g. Cohen's *d*, Pearson's *r*), indicating how they were calculated |

*Our web collection on statistics for biologists contains articles on many of the points above.*

## Software and code

Policy information about availability of computer code

| | |
|---|---|
| Data collection | Zoo system (X-ray), SerialEM 3.8 (cryoEM) |
| Data analysis | KAMO, XDS (March 15, 2019), ccp4 (7.0.078), PHENIX 1.18, Refmac5, Relion3.1, MotionCorr2.1, CTFFIND4, Coot(0.9.2), PYMOL(2.3.1), Chimera(1.14), ChimeraX (1.2.2), GraphPad PRISM 4, CLUSTALW, |

For manuscripts utilizing custom algorithms or software that are central to the research but not yet described in published literature, software must be made available to editors and reviewers. We strongly encourage code deposition in a community repository (e.g. GitHub). See the Nature Portfolio guidelines for submitting code & software for further information.

## Data

Policy information about availability of data

All manuscripts must include a data availability statement. This statement should provide the following information, where applicable:

- Accession codes, unique identifiers, or web links for publicly available datasets
- A description of any restrictions on data availability
- For clinical datasets or third party data, please ensure that the statement adheres to our policy

Atomic coordinates and a cryo EM density map reported in this study have been deposited in the Protein Data Bank (http://www.rcsb.org) and the Electron Microscopy Data Bank (https://ebi.ac.uk/pdbe/emdb/) as follows;
2ZXE : Crystal structure of the sodium - potassium pump in the E2.2K+.Pi state
4RES : Crystal structure of the Na,K-ATPase E2P-bufalin complex with bound potassium
5YLV : Crystal structure of the gastric proton pump complexed with SCH28080
6JXH : K+-bound E2-MgF state of the gastric proton pump (Tyr799Trp)

7EFL : Crystal structure of the gastric proton pump K791S mutant in Rb+-bound (BYK)E2BeF state
7EFM: Crystal structure of the gastric proton pump K791S/E820D/Y340N mutant in Rb+-bound (BYK)E2BeF state
7EFN: Crystal structure of the gastric proton pump K791S/E820D/Y340N/E936V mutant in Rb+-bound (BYK)E2BeF state
7ET1: Cryo-EM structure of the gastric proton pump K791S/E820D/Y340N/E936V/Y799W mutant in K+-occluded (K+)E2-AlF state
EMDB-31294 : Cryo-EM structure of the gastric proton pump K791S/E820D/Y340N/E936V/Y799W mutant in K+-occluded (K+)E2-AlF state

# Field-specific reporting

Please select the one below that is the best fit for your research. If you are not sure, read the appropriate sections before making your selection.

☒ Life sciences ☐ Behavioural & social sciences ☐ Ecological, evolutionary & environmental sciences

For a reference copy of the document with all sections, see nature.com/documents/nr-reporting-summary-flat.pdf

# Life sciences study design

All studies must disclose on these points even when the disclosure is negative.

| | |
|---|---|
| Sample size | No statistical method was used to determine sample size. Functional experiments were performed multiple times with similar results and further inclusion of data did not change the results. Complete X-ray and cryoEM statistics are provided in supplementary Table 3 and 4. |
| Data exclusions | Data selection for cryo-EM is illustrated in supplementary table 3 and supplementary fig 3. For other experiment, no data were excluded from the analysis. |
| Replication | Biochemical assays, including protein expression, thermal stability and ATPase activity were examined at least three times independently, and their average or representative ones were shown in the manuscript |
| Randomization | Randomization is not relevant for this study, as there were no groups allocated in any of the experiments |
| Blinding | Not applicable as this is deemed not practically feasible |

# Reporting for specific materials, systems and methods

We require information from authors about some types of materials, experimental systems and methods used in many studies. Here, indicate whether each material, system or method listed is relevant to your study. If you are not sure if a list item applies to your research, read the appropriate section before selecting a response.

## Materials & experimental systems

| n/a | Involved in the study | |
|---|---|---|
| ☐ | ☒ | Antibodies |
| ☐ | ☒ | Eukaryotic cell lines |
| ☒ | ☐ | Palaeontology and archaeology |
| ☒ | ☐ | Animals and other organisms |
| ☒ | ☐ | Human research participants |
| ☒ | ☐ | Clinical data |
| ☒ | ☐ | Dual use research of concern |

## Methods

| n/a | Involved in the study | |
|---|---|---|
| ☒ | ☐ | ChIP-seq |
| ☒ | ☐ | Flow cytometry |
| ☒ | ☐ | MRI-based neuroimaging |

## Antibodies

| | |
|---|---|
| Antibodies used | anti-FLAG M2 affinity resin, no dilution |
| Validation | https://www.sigmaaldrich.com/catalog/CertOfAnalysisPage.do?symbol=A2220&LotNo=SLBT8835&brandTest=SIGMA&returnUrl=%2Fproduct%2FSIGMA%2FA2220 |

## Eukaryotic cell lines

Policy information about cell lines

| | |
|---|---|
| Cell line source(s) | HEK293S GnT1- (ATCC CRL-3022) |
| Authentication | No further authentication was performed for cell lines. |
| Mycoplasma contamination | Not tested for mycoplasma contamination. |


March 2021

| | |
|---|---|
| Commonly misidentified lines<br>(See ICLAC register) | No commonly misidentified cell lines were used. |
